# Histomorphometric Evaluation of Cartilage Degradation using Rabbit Articular Chondrocytes Cultured in Alginate Beads − Effects of Hyaluronan

**Published:** 2010-06

**Authors:** K. Nakatsuka, K. Kurita, Taro Hayakawa, Katsuhito Nakashima, Kyoko Yamashita, Takeshi Hoshino, Kyosuke Miyazaki

**Affiliations:** 1 *Department of Oral and Maxillofacial Surgery, School of Dentistry, Aichi-Gakuin Unuverity, Japan;*; 2 *Department of Biochemistry, School of Dentistry, Aichi-Gakuin Unuverity, Japan;*; 3 *Seikagaku Corporation, Japan*

**Keywords:** chondrocyte, matrix metalloproteinases, alginate beads, proteoglycan

## Abstract

**Objective:**

A 3-dimensional alginate bead culturing method using rabbit articular chondrocytes was studied for the screening of the effectiveness of drugs for articular diseases.

**Design:**

The beads cultured with IL-1β, TGF-β, and Hyaluronan (HA) were evaluated histochemically with Alecian blue and immunohistochemically with CS-56 antibody. Chondrocytes in alginate beads were arbitrarily classified into four groups: 1) chodrocyte surrounded with cell-associated matrix (CAM) in which proteoglycan (PG) was positively stained (PG-possitive chondrocyte); 2) chondrocyte with PG-negative CAM; 3) PG-positive CAM alone, and 4) PG-negative CAM alone. Total sulfated GAG concentrations in the culture media were quantitated by dimethylmethylene blue (DMMB) assay. ProMMP-3, TIMP-1 and –2 concentrations in the culture media were determined by sandwich enzyme immunoassays.

**Results:**

Significant increase of PG-nagative cells were immunohistochemically found by IL-1β stimulation. The pretreatment with TGF-β almost fully suppressed those increase of PG-negative cells by IL-1β. Both GAG and proMMP-3 concentrations in the culture media were significantly increased after IL-1β stimulation. There were no significant differences in both TIMP-1 and TIMP-2 concentrations in the culture media with or without IL-1β stimulation. 800-kDa HA reduced significantly the number of PG-negative cells and proMMP-3 concentration in the culture media, but showed no effects on the concentrations of both TIMPs.

**Conclusions:**

Because this 3-dimensional chondrocyte culture in alginate beads is close to *in vivo* conditions, this method can be used for evaluation of the effectiveness of novel drugs for articular diseases.

## INTRODUCTION

The major components of the extracellular matrix (ECM) of articular chondrocytes are collagen and proteoglycan. In articular diseases such as osteoarthrosis (OA) and chronic rheumatoid arthritis (RA), the destruction of these ECM components is a factor of articular dysfunction (1, 2). In articular cartilage destruction, various proteinases are involved. In particular, matrix metalloproteinases (MMP) (3-9), represented by MMP-3 and MMP-13, aggrecanses (10, 11), such as ADAMTS-4 and ADAMTS-5, and tissue inhibitors of matrix metalloproteinases (TIMP) (12-16), known as MMP inhibitors, have been suggested to play important roles. Previous studies have shown the expression of MMP, ADAMTS-4, and ADAMTS-5 after Interleukin-1β (IL-1β) stimulation, degradation of cartilage ECM components due to their expression (17-20), and the inhibition of this degradation by transforming growth factor-β1 (TGF-β) stimulation (21). These proteinases have also been detected at high concentrations in the synovial fluid of patients in the early stage of OA or RA (22). However, the mechanism of ECM degradation and the detailed role of each proteinase remain unclear.

Many basic studies have been performed on cartilage tissue destruction using articular chondrocytes, mostly by monolayer culture. Chondrocytes in monolayer cultures are known to undergo dedifferentiation, transforming into fibroblast-like cells. Therefore, results obtained under the monolayer culture condition do not always reflect the original character of chondrocytes.

**Figure 1:**
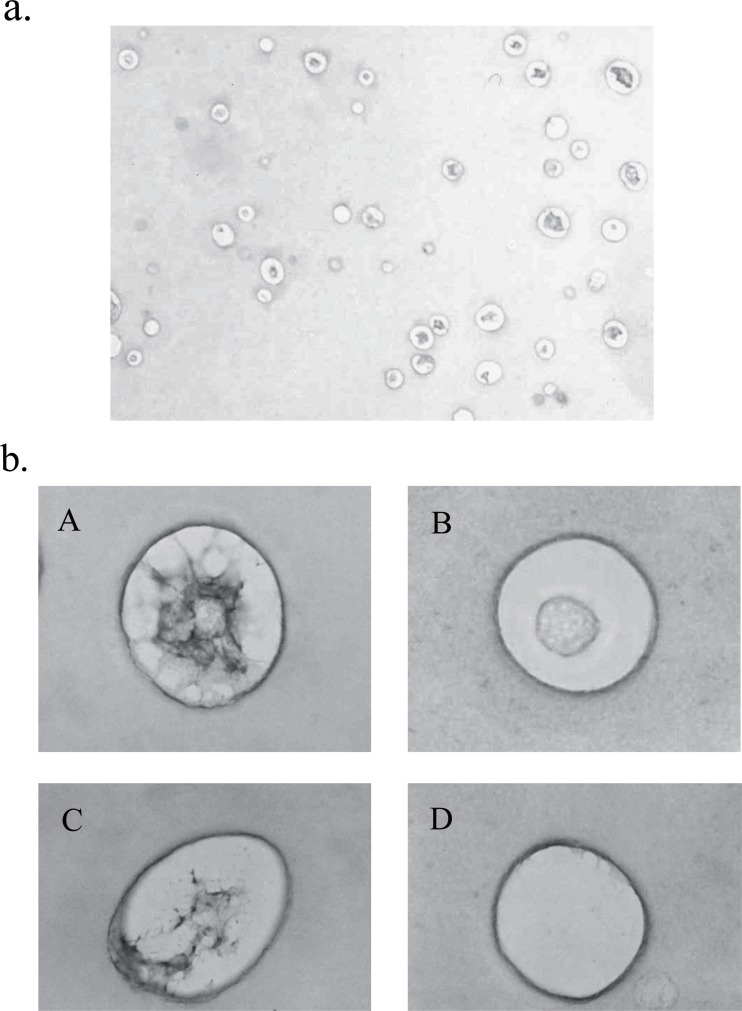
Alcian blue staining of the section of rabbit articular chondrocytes on day 5 in alginate beads (a), and a variety of cellular characteristics within the section (b). A, PG-positive chondrocyte; B, PG-negative chondrocyte; C, PG-positive cell-associated matrix (CAM) alone; D, PG-negative CAM alone.

In other studies, chondrocytes were 3-dimensionally cultured in an environment closer to the in vivo environment using various scaffolds (23). As 3-dimensional chondrocyte culture types, agar culture, culture in collagen, high-density tube culture, and agarose culture have long been performed. In recent years, culture using alginate as a glycuronan derived from brown seaweed algae has attracted attention. Culture in alginate beads is considered to be advantageous for in vivo studies of chondrocytes because this method allows long-term culture while maintaining the original character of chondrocytes, and facilitates the isolation and recovery of chondrocytes by the solution of alginate with EDTA (24) (Fig. 1).

With this background, to confirm the previous in vivo findings, we performed 3-dimensional culturing of rabbit articular chondrocytes in alginate beads, and evaluated the influences of IL-1β and TGF-β. These results show the possibility that this alginate bead culture method can support the screening of the effectiveness of novel drugs for articular diseases. Therefore, we re-evaluated hyaluronan (hyaluronic acid, HA) among clinically used drugs for articular diseases.

## METHODS

### Materials

Reagents used and their sources were as follows: Dulbecco’s modified Eagle’s medium (DMEM) from Gibco Laboratories; actinase from Kaken Pharmaceutical; bacterial collagenase (*Clostridium histolyticum*) from Worthington; alcian blue 8GX from Sigma; toluidine blue-O from Sigma; anti-chondroitin sulfate(CS-56) antibody from Seikagaku Corporation; interleukin-1β (IL-1β) and transforming growth factor-β1 (TGF-β) from Roche Diagnostics; 3, 30, 200 and 500-kDa hyaluronan (HA) from Fidia S. p. A.; 800 and 2,300-kDa HA from Seikagaku Corporation.

### Chondrocyte culture in alginate beads

Full thickness articular cartilage slices were aseptically collected from the shoulder joints of young Japanese white rabbits (female, ca. 1 kg weight). Chondrocytes were released from the slices by sequential digestion of the tissue with 0.4% actinase and 0.025% bacterial collagenase at 37°C, for 14 h. The isolated cells were suspended at a density of 1 × 10^6^ cells/ml in the sterile alginate solution (1.2% alginate in 0.15 M NaCl). All the HA preparations were added into cell suspension before polymerizing into beads. Final concentration of HA was 100 μg/ml unless otherwise indicated. The cell suspension was then slowly expressed through a 22-gauge needle in a dropwise fashion into a 102 mM CaCl_2_ solution, and the beads were allowed to polymerize for 10 min under gentle stirring. After three consecutive washes with 10 volumes of 0.15 M NaCl solution, the beads were incubated for 5 days at 37°C in a humidified atmosphere of 5% CO_2_. The beads were then transferred into FCS-free DMEM and incubated for 24 h, and then stimulated with IL-1β or TGF-β alone for 48 h, or IL-1β for 48 h following the 6 h-pretreatment with TGF-β.

### Histochemistry and immunohistochemistry

Alginate beads were fixed in 4% paraformaldehyde for 4 h. After being embedded in paraffin, 5 μm sections were stained with Alcian blue or toluidine blue. For immunohistochemical evaluation, unstained 5 μm sections were deparaffinized and rehydrated. Endogenous peroxidase activity was quenched by immersion in 3% H_2_O_2_ in methanol. After non-specific binding was blocked with normal blocking serum. Slides were incubated overnight at 4°C with anti-chondroitin sulfate (CS-56) (Seikagaku Corporation) and anti-TIMP-3 monoclonal antibody (Daiichi Fine Chemical). Following day, the slides were incubated with biotinylated secondary antibody. Bound primary antibody was detected using an avidin-horseradish peroxidase method with diaminobenzidine (DAB) chromogenic substrate.

One hundred and fifty cells were counted on each section by three investigators, who were unaware of experimental conditions. The percentages of the number of PG-negative cells were calculated, and the values were expressed in mean ± SD (n-3).

### Glycosaminoglycan assay

Total sulfated GAG concentrations in the culture media were quantitated by dimethylmethylene blue (DMMB) assay (Farndale *et al*., 1986), and reported in μg/ml.

### Precursor form of matrix metalloproteinase-3, tissue inhibitor of metalloproteinases-1 and -2 assays

ProMMP-3, TIMP-1 and -2 concentrations in the culture media were determined by sandwich enzyme immunoassays for rabbit proMMP-3, TIMP-1 and TIMP-2. Rabbit TIMP-1 and -2 concentrations were calculated by using standard curves for human TIMP-1 and -2, respectively, assuming that they both had the same affinity for the monoclonal antibodies.

## RESULTS

### Histochemical and immunohistochemical studies

**Figure 2:**
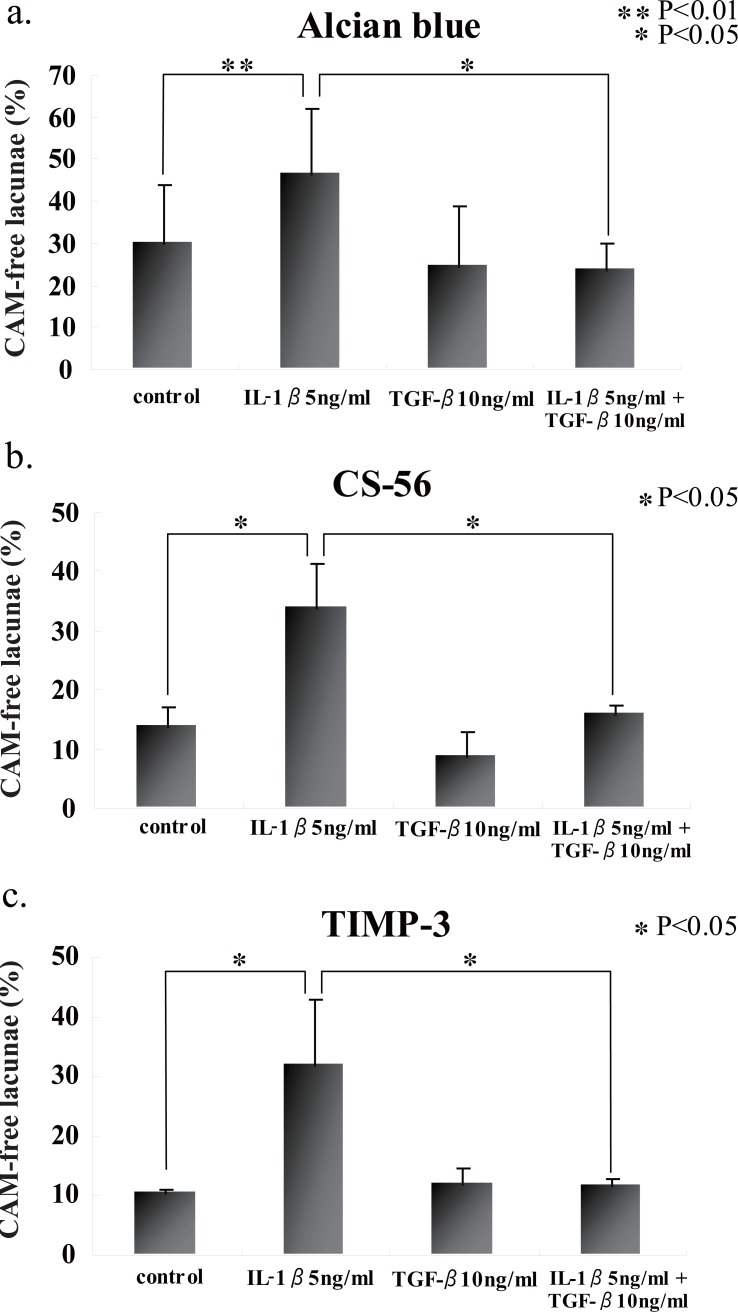
Percentages of PG-negative chondrocytes in alginate beads stained with Alcian blue or CS-56 or anti-TIMP-3 antibody under different conditions. The number of PG-negative cells in 150 cells was significantly increased in the beads stimulated with IL-1β. TGF-β, however, prevented the loss of PG. Values are mean ± SD (n=3). *p<0.05, **p<0.01.

We first stained a section of rabbit articular chondrocytes in alginate beads on day 5 with Alcian blue, and found a variety of cellular characteristics (Fig. 1a). They were arbitrarily classified into four groups (Fig. 1b). Namely, chodrocyte surrounded with cell-associated matrix (CAM) in which proteoglycan (PG) was positively stained (PG-possitive chondrocyte) (A), chondrocyte with PG-negative CAM (B), PG-positive CAM alone (C), and PG-negative CAM alone (D). We counted 150 cells and founded that about 30 percent of them had PG-negative CAM. This finding was almost consistent with the section stained with toruidine blue (data not shown). We exposed the cells in beads to IL-1β for 48 h and found the significant increase of PG-negative cells upto 40-50%. Immunohistochemical study with CS-56 antibody showed more significant difference between cells with or without IL-1β stimulation (Fig. 2b). The 6 h-pretreatment with TGF-β almost fully suppressed those increase of PG-negative cells by IL-1β (Fig. 2a and b).

### Gag and prommp-3 concentrations in the culture media of alginate beads

**Figure 3:**
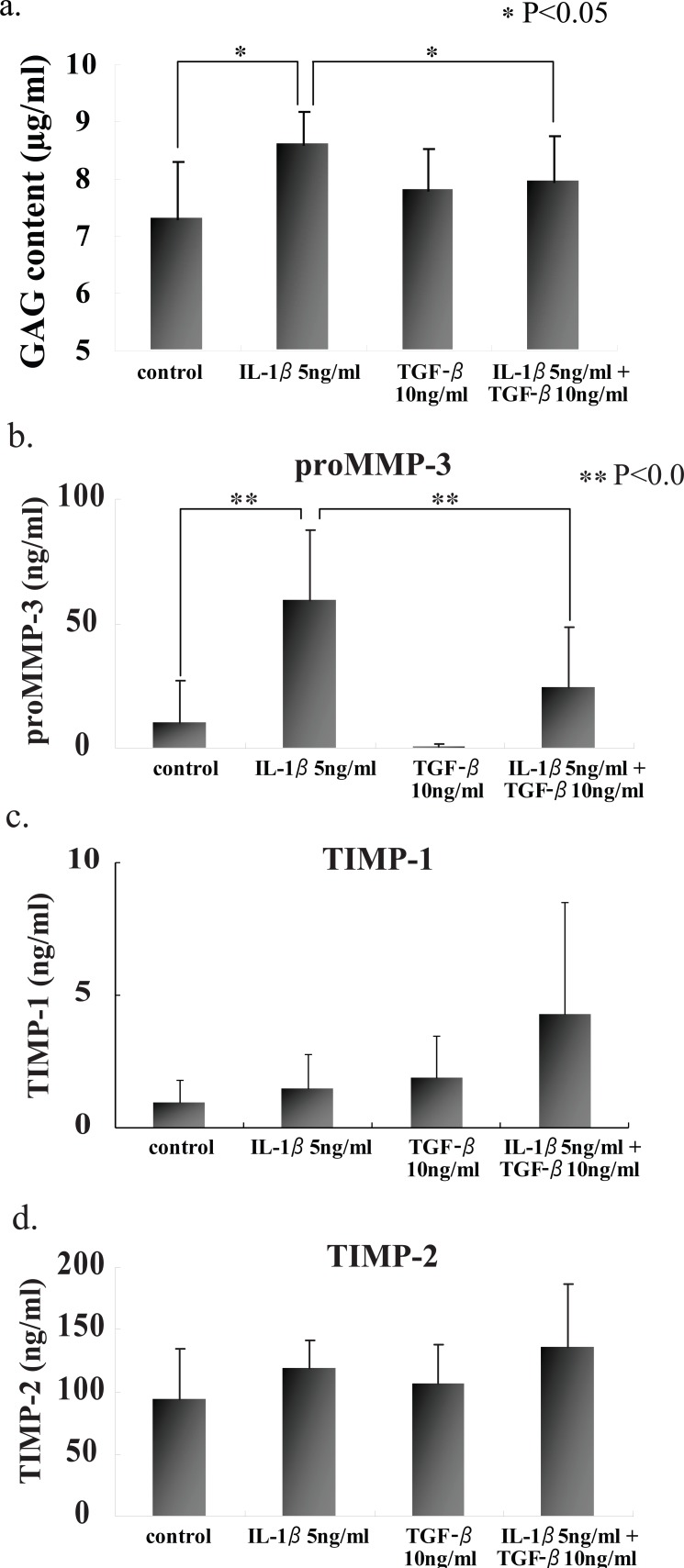
GAG, proMMP-3, TIMP-1 and –2 contents in the culture media of alginate beads under different conditions. GAG and proMMP-3 contents were significantly increased in the culture media of beads stimulated with IL-1β. TGF-β, however, suppressed the incease of those contents. Values are mean ± SD (n=3). *p<0.05, **p<0.01.

In attempt to elucidate the histochemical findings with respect to the degradation of PG in CAM, GAG and proMMP-3 concentrations were determined in the culture media with or without IL-1β stimulation. Both GAG and proMMP-3 concentrations in the culture media were significantly increased after IL-1β stimulation (Fig. 3a and b). Again pretreatment with TGF-β prevented the release of both GAG and proMMP-3 into the culture media. Those results were consistent with histochemical findings. Although there were no significant differences in both TIMP-1 and TIMP-2 concentrations in the culture media with or without IL-1β stimulation (Fig. 3c and d).

### Effects of hyaluronan on the gag release into the culture media of beads stimulated with il-1β

**Figure 4:**
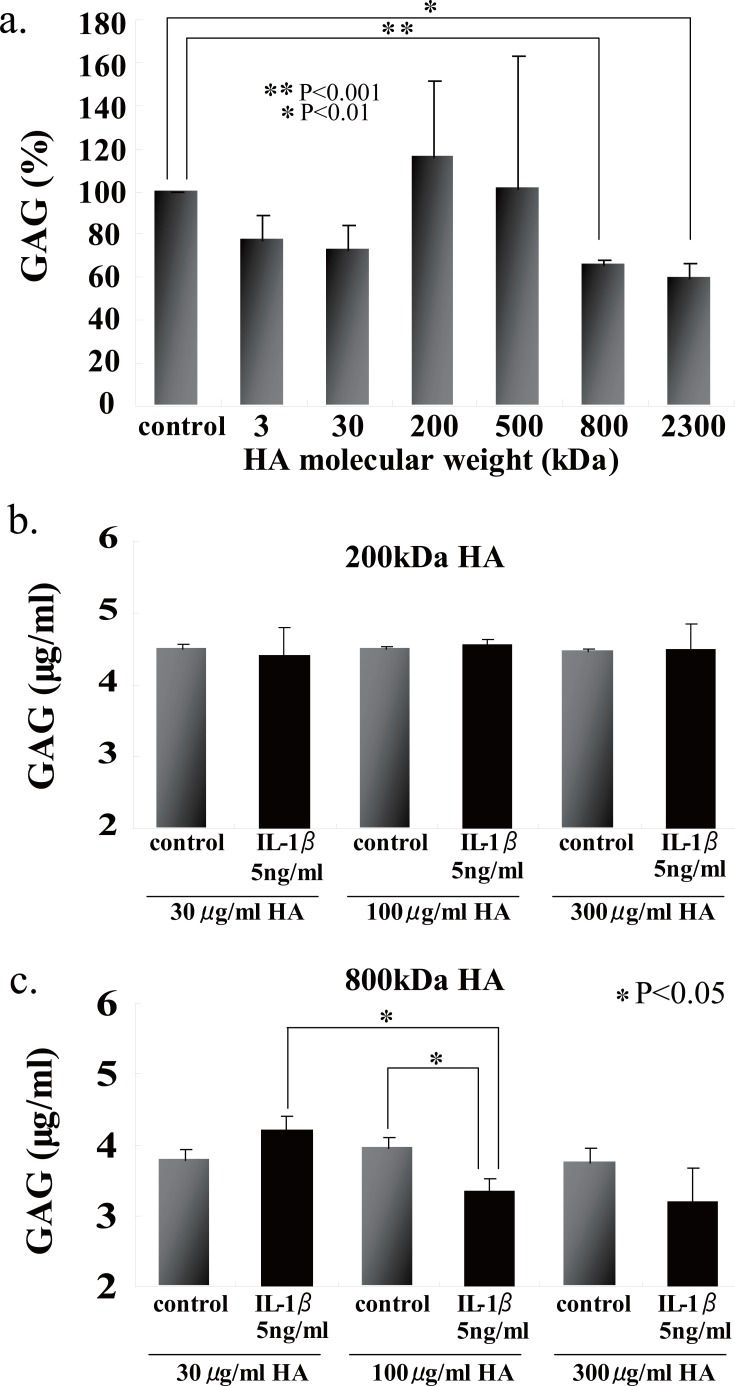
Effects of various sizes of HA (a) and the concetrations of 200(b)- and 800-kDa HA (c) on the release of free GAG into the culture media of alginate beads stimulated with IL-1β. a, HA concentrations were all 100 μg/ml. Both 800- and 2300-kDa HAs significantly suppressed the release of GAG. c, only 800-kDa HA at 100 μg/ml significantly suppressed the release of GAG from beads stimulated with IL-1β. Values are mean ± SD (n=3). *p<0.05, **p<0.01, ***p<0.001.

**Figure 5:**
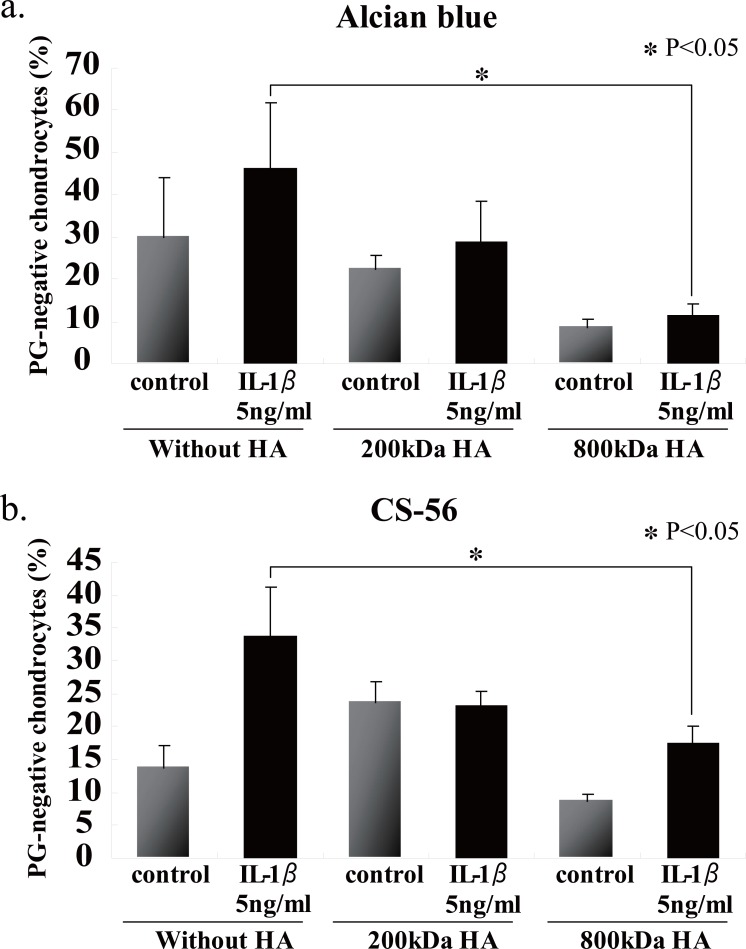
Effects of 200- or 800-kDa HA on the percentages of PG-negative chondrocytes in alginate beads stimulated with IL-1β. Only 800-kDa HA significantly suppressed the release of GAG stimulated with IL-1β. Values are mean ± SD (n=3). *p<0.05.

**Figure 6:**
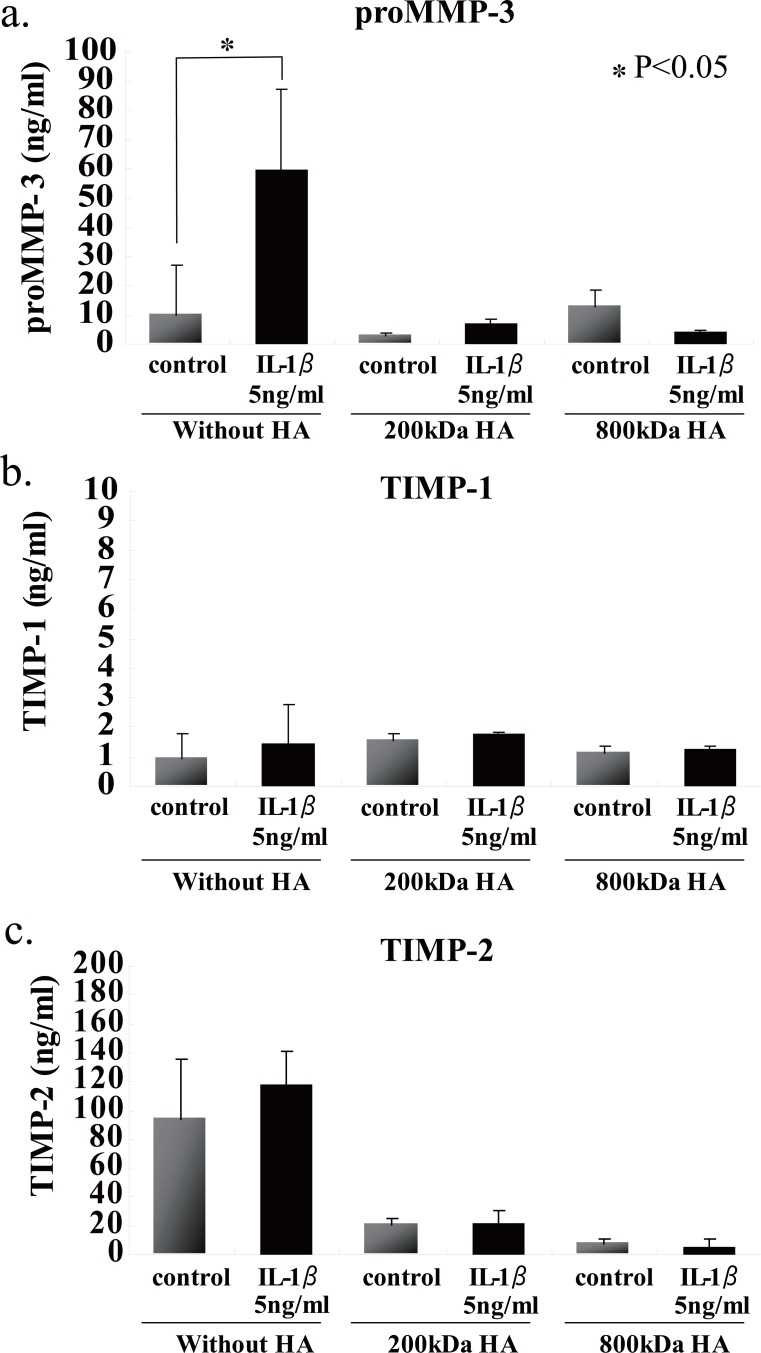
Effects of 200- or 800-kDa HA on proMMP-3, TIMP-1 or –2 contents in alginate beads stimulated with IL-1β. Either 200- or 800-kDa HA significantly suppressed the release of proMMP-3, but essentially no effect on the release of TIMPs. Values are mean ± SD (n=3). *p<0.05, **p<0.01.

We first examined the effects of HA sizes on the GAG release into the culture media, and found that two HAs having high molecular mass, i.e., 800- and 2300-kDa HAs, were significantly suppressed GAG release from the beads (Fig. 4a). Choosing 200- and 800-kDa HAs as ineffective and effective examples for the prevention of GAG release, respectively, we checked the effects of HA concentrations on the release of GAG into the culture media, and then found that 200-kDa HA did not show any significant difference among three different concentrations (Fig. 4b), and only 800-kDa HA at 100 μg/ml significantly suppressed the release of GAG (Fig. 4c). In an attempt to elucidate the findings on GAG release with respect to the cellular characteristics, the number of PG-negative cells were counted in the section of beads stimulated with IL-1β in the presence or absence of 200- and 800-kDa HAs. Again only 800-kDa HA reduced significantly the number of PG-negative cells in the sections stained with either Alcian blue or CS-56 antibody (Fig. 5). Furthermore, we examined the effects of 200- and 800-kDa HAs on the release of proMMP-3, TIMP-1 and TIMP-2 into the culture media of beads stimulated with IL-1β, and found that both HAs reduced significantly rise of proMMP-3 concentration in the culture media stimulated by IL-1β (Fig. 6a), but showed no effects on the concentrations of both TIMPs (Fig. 6b and c).

## DISCUSSION

Articular cartilage tissue is an avascular and relatively simple tissue composed of chondrocytes and ECM. Cartilage tissue destruction observed in diseases such as OA and RA is primarily the degradation of ECM components. ECM components slightly vary according to the location of the articular cartilage and its depth from the surface, but major components are collagen and aggrecan as a proteoglycan. Fibrous type II collagen forms a network in which aggrecan with high water retention is trapped. In articular diseases such as OA and RA, various proteinases represented by MMP-3, MMP-13, and aggrecaneses are directly involved in the destruction of cartilage ECM.

The expression of these proteinases is induced by inflammatory cytokines such as IL-1, resulting in the degradation of proteoglycans of mainly aggrecan and type II collagen. Previous studies using the culture system of articular cartilage tissues of various animals have already suggested tissue destruction that appears to represent the disappearance of aggrecan after IL-1 stimulation. This study using rabbit articular cartilage tissue confirmed similar tissue destruction after IL-1β stimulation.

In addition, TGF-β has been suggested to not only promote the production of cartilage ECM (28) but also play a defensive role in cartilage tissue destruction observed in various diseases (29). However, opposite results such as the promotion of MMP-13 production (30) and the degradation of cartilage ECM (31) by TGF-β have also been reported, and therefore, the role of TGF-β is still unclear. In this study, stimulation with TGF-β alone resulted in the slight disappearance of proteoglycan on the surface layer of articular cartilage tissue. However, TGF-β coexisting with IL-1β markedly inhibited IL-1β-induced cartilage tissue destruction. These findings on histological staining were well reflected by the content of GAG released into the culture supernatant. TGF-β coexisting with IL-1β markedly inhibited an increase in GAG due to proteoglycan degradation by IL-1β. These results suggest the defensive role of TGF-β in articular cartilage destruction induced by IL-1β.

There have been various basic studies on chondrocytes following monolayer culture. Chondrocytes after monolayer culture undergo character transformation, showing fibroblast-like characteristics, and produce ECM components such as type I collagen that is not generally produced by chondrocytes. After 3-dimensional chondrocyte culture in alginate beads, such transformation is not observed, and chondrocytes proliferate while maintaining the round shape proper to chondrocytes, and preserve ECM components, mainly type II collagen and aggrecan, characteristic of chondrocytes, even after long-term culture for some months (27). Previous studies by 3-dimensional culture in alginate beads have suggested that chondrocytes can be cultured under conditions close to the in vivo conditions. Therefore, in this study, we morphologically evaluated chondrocytes on the 7th day of culture in alginate beads by Alcian blue and toluidine blue staining, and observed a few variations in the morphology of lacunae formed around chondrocytes by each staining. These findings have not been clearly described before, and so are novel. We don’t understand the reason for these variations in the morphology, since chondrocytes forming and maintaining each lacunae do not synchronize with those forming other lacunae. The variations may reflect differences in the phase of the cell cycle.

On the other hand, among variations in the morphology of lacunae, we focused on lacunae with unstained CAM, indicating the disappearance of CAM. The percentage of such lacunae markedly increased in the group stimulated by IL-1β alone. However, the coexistence of TGF-β with IL-1β decreased this percentage to the control level. Similar results were obtained after immunostaining with anti-CS-56 or anti-TIMP-3 antibody, i.e., irrespective of the staining method. These results together with the above obtained in cultures of the same rabbit articular cartilage tissues suggest that the disappearance of CAM in lacunae in alginate beads is due to the degradation of proteoglycan. The increase in the percentage of lacunae lacking CAM after IL-1β may reflect cartilage tissue destruction.

Among the 4 types of TIMP previously reported, TIMP-3 alone binds to ECM (32, 33) and specifically binds to sulfated GAG such as chondroitin sulfate via its N terminal (34). Unlike the other types of TIMP, TIMP-3 inhibits ADAM (35-37) and ADAMTS (10, 11) MMP. As shown in Figs. 2, the distribution pattern of 5 morphological types of lacunae classified after the staining of chondroitin sulfate with anti-CS-56 antibody was well consistent with that classified following staining with anti-TIMP-3 antibody. As described above, assuming that chondroitin sulfate is bound to TIMP-3, the two act together, which may provide a good explanation of the above results, though the explanation has not yet been confirmed by the double staining method.

The mechanism of TGF-β defense against the IL-1β-induced cartilage ECM destruction is still unclear. Washimi et al reported the specific enhancement of TIMP-3 mRNA expression by TGF-β in rabbit articular chondrocytes after 3-dimensional culture in alginate beads. It is also possible that TIMP-3 expressed by such a mechanism binds to proteoglycan via chondroitin sulfate, inhibiting degradation not only by MMP but also by aggrecanase, as described above.

As for the effects on cartilage degradation of the hyaluronan, various reports have been performed so far. It has been especially proven to the transformable cartilage to have the action of biphasic. Moreover, IL-1 controls the inducement of TIMPs, and the report of recovering this action is performed as for the hyaluronan.

Three-dimensional culture in alginate beads is useful for in vitro studies on cartilage tissue destruction and allows the numerical expression of the degree of destruction. In this aged society, the number of patients with articular diseases such as OA or RA has been increasing, and the main characteristic of these diseases is articular cartilage destruction. Drugs for defense against this destruction are needed. It is possible that the disappearance of CAM in lacunae and its recovery described above can contribute to the evaluation of the effectiveness of novel drugs. At present, we are evaluating this possibility for drugs clinically used.

In conclusion, the disappearance of CAM after IL-1β stimulation and its inhibition by coexisting TGF-β, were observed after 3-dimensional chondrocyte culture in alginate beads. Each lacunae observed in 3-dimensional chondrocyte culture in alginate beads may reflect responses of cartilage tissue to inflammatory cytokines and can be regarded as the minimum unit of cartilage tissue. These results suggest that 3-dimensional chondrocyte culture in alginate beads allows studies on articular cartilage destruction under conditions close to the in vivo conditions and can be used for evaluation of the effectiveness of novel drugs for articular diseases.
